# Fiber Bragg Grating Sensors for Performance Evaluation of Fast Magnetic Resonance Thermometry on Synthetic Phantom

**DOI:** 10.3390/s20226468

**Published:** 2020-11-12

**Authors:** Martina De Landro, Jacopo Ianniello, Maxime Yon, Alexey Wolf, Bruno Quesson, Emiliano Schena, Paola Saccomandi

**Affiliations:** 1 Department of Mechanical Engineering, Politecnico di Milano, via Giuseppe La Masa 1, 20156 Milan, Italy; paola.saccomandi@polimi.it; 2 Unit of Measurements and Biomedical Instrumentation, Departmental Faculty of Engineering, Università Campus Bio-Medico di Roma, via Alvaro del Portillo 21, 00128 Rome, Italy; jacopo.ianniello@alcampus.it (J.I.); e.schena@unicampus.it (E.S.); 3 Institut Hospitalo-Universitaire, Liryc Institut de Rythmologie et Modélisation Cardiaque, Avenue du Haut Lévêque, 33600 Pessac, France; maxime.yon@ihu-liryc.fr (M.Y.); bruno.quesson@ihu-liryc.fr (B.Q.); 4 Laboratory of Fiber Optics, Institute of Automation and Electrometry of the SB RAS, 1 Acad. Koptyug Ave., 630090 Novosibirsk, Russia; wolf@iae.nsk.su

**Keywords:** thermometry, fiber bragg grating sensors, magnetic resonance thermometry imaging, accuracy evaluation, gradient-echo echo-planar imaging, laser ablation

## Abstract

The increasing recognition of minimally invasive thermal treatment of tumors motivate the development of accurate thermometry approaches for guaranteeing the therapeutic efficacy and safety. Magnetic Resonance Thermometry Imaging (MRTI) is nowadays considered the gold-standard in thermometry for tumor thermal therapy, and assessment of its performances is required for clinical applications. This study evaluates the accuracy of fast MRTI on a synthetic phantom, using dense ultra-short Fiber Bragg Grating (FBG) array, as a reference. Fast MRTI is achieved with a multi-slice gradient-echo echo-planar imaging (GRE-EPI) sequence, allowing monitoring the temperature increase induced with a 980 nm laser source. The temperature distributions measured with 1 mm-spatial resolution with both FBGs and MRTI were compared. The root mean squared error (RMSE) value obtained by comparing temperature profiles showed a maximum error of 1.2 °C. The Bland-Altman analysis revealed a mean of difference of 0.1 °C and limits of agreement 1.5/−1.3 °C. FBG sensors allowed to extensively assess the performances of the GRE-EPI sequence, in addition to the information on the MRTI precision estimated by considering the signal-to-noise ratio of the images (0.4 °C). Overall, the results obtained for the GRE-EPI fully satisfy the accuracy (~2 °C) required for proper temperature monitoring during thermal therapies.

## 1. Introduction

Thermal therapies are used to induce cell coagulative necrosis leading to tissue alterations at both conformational and metabolic levels [1]. In tumor treatment, the temperature increment needs to be localized into the target, while leaving the surrounding healthy tissue unhurt [2]. To allow optimal therapy outcome with minimally invasive thermal treatments, real-time temperature monitoring of the tissue is of great interest [3]. Indeed, for a given local emitted energy, the resulting tissue thermal damage depends on effective temperature-time evolution which is closely linked to tissue composition and physiological parameters [4]. As a result, the actual tissue temperature and the subsequent thermal damage are difficult to predict suing emitted energy as a single input. Hence, continuous thermal mapping during treatment is preferred to better monitor and control the therapeutic outcomes of the procedure. Commercial systems for radiofrequency ablation and microwave ablation are provided with needles embedding one or a few temperature sensors (e.g., thermocouples), allowing the temperature of the tissues to be measured in contact with the needle [5,6]. However, these sensors cannot furnish temperature mapping of the tissues surrounding the heating device thus providing limited information to the surgeon. Due to local differences in heat conduction in biological media, the procedure implementation would benefit from the two or three-dimensional information of the tissue temperature. Magnetic Resonance Thermometry Imaging (MRTI) is the most promising technic allowing multidimensional in vivo temperature mapping, as well as target definition [7]. MR temperature estimation dates back to 1948 when Bloembergen et al. [8] first studied the correlation of MR parameters with temperature. Nevertheless, it is only in 1983 that Parker and colleagues [9] started to investigate its potential as a non-invasive approach to monitor the internal temperature. The sensitivity to temperature variations is demonstrated by different MR parameters, such as T_1_ and T_2_* relaxation times, the water proton resonance frequency shift (PRFS), and the diffusion coefficient [10]. Nowadays, the most widely used MRTI method is the PRFS due to its excellent linearity from 0 °C to 100 °C, its near-independence with respect to tissue type (except for adipose tissues which lacks in the necessary oxygen-hydrogen-bound), and its good temperature sensitivity [7,11,12]. PRFS method is compatible with all the MR-compatible heating devices [12].

Currently, MRTI is largely employed during thermal treatments such as laser ablation and focused ultrasound [10,13]. Laser ablation is especially well-suited for MR guidance since the thin fiber optic applicator does not create large susceptibility artifacts in the MR images, and a relatively small field of view is sufficient to monitor the localized heating around the fiber [14]. The efficacy of MRTI-guidance during a thermal therapy depends on: (1) Excellent spatial (1–3 mm) and temporal resolution (1 s or less); (2) robustness to artifacts; (3) accuracy of temperature values (better than 1–2 °C) [15]. Owing to its robustness and easy setting, the gradient echo (GRE) pulse sequences currently remain the most used for PRFS MRTI clinical study [10]. Recent studies have shown that fast temperature mapping can be achieved with the gradient-echo echo-planar imaging (GRE-EPI) sequence. Due to its fast acquisition of all the k-space lines in tens of milliseconds following a single radiofrequency excitation pulse, single-shot EPI provides a much better temporal resolution than the GRE sequence, which can lead to a more accurate temperature determination [16] and higher robustness to motion [17]. Although, in providing fast temperature mapping, the EPI technical requirements and sensitivity to artifacts have first restricted its use in clinical practice [18]. Indeed, the low bandwidth in the EPI phase dimension may induce spatial distortion and its inherent long echo time can also lead to important signal attenuation in tissue with short T_2_* [19]. To mitigate these artifacts, EPI train length can be reduced using high gradient strength combined with various acceleration methods such as GRAPPA (GeneRalized Autocalibrating Partial Parallel Acquisition) and/or partial Fourier [20,21], at the cost of a slight reduction of image signal to noise (SNR) altering temperature precision. Although the GRE-EPI PRFS method is prone to several artifacts [10], it is increasingly used to monitor ablations clinically, since rapid imaging techniques allow volumetric imaging of temperature distribution in a few seconds, thereby allowing direct visualization of the heating [22]. To evaluate the accuracy of GRE-EPI, while avoiding bias, due to localized magnetic field inhomogeneity it is important to use multiple and susceptibility-matched sensors such as Fiber Bragg Grating (FBG) sensors.

In this article, the accuracy and precision of the GRE-EPI thermometry were evaluated through FBGs with a millimetric spatial resolution during laser-induced heating. This comparison allows the temperature bias between FBG and GRE-EPI thermometry to be measured in numerous spatial locations. Temperature uncertainty due to the SNR of the magnitude image usually used to estimate the precision of the MRTI was also measured [18,23,24]. The SNR method accounts for T_1_ increase and intra-voxel dephasing; both effects will decrease the signal and thus the SNR. FBGs can be safely used during MR-guided thermal procedures because they are immune to electromagnetic interference [25,26,27]. In contrast to temperature electrical sensors, FBGs are not prone to temperature overestimation due to their material (glass or polymer) and have low heat conductivity. Both properties are crucial to avoid any measurement errors, particularly when FBGs are used under laser irradiation. Moreover, a single array of FBGs can perform multi-point measurements simultaneously by inserting a single small-sized element (i.e., an optical fiber with an outer diameter of hundreds of micrometers) enabling the reconstruction of the temperature profile with accuracy for each sensor of 0.1 °C. Nowadays, the use of FBGs for temperature monitoring of tissues undergoing thermal treatments has been largely investigated [5,28,29,30]. However, some drawbacks in these applications are the cross-sensitivity to strain and the invasiveness [31]. In relation to the combined use of FBGs in the MR imaging scenario, some research groups have proposed FBG-based sensors for monitoring temperature as either additional support during thermal therapies [32,33,34,35] or reference system for MRT accuracy evaluation [36].

In our study, the performance of MRTI employing a GRE-EPI sequence were evaluated. Firstly, a standard approach based on the SNR of MR images was employed to estimate the MRTI precision. Secondly, MRTI accuracy was assessed during a laser ablation in 3% agar gel using temperature measurements provided by 1 FBG/mm spatially resolved fiber optic sensors used as reference. One fiber housing 25 FBGs was inserted in the gel and employing a high-resolution image, a pixel-FBG sensor analysis was performed. Comparisons between the FBG and MRTI measurements was performed extracting a root mean square error (RMSE) between the two temperature space lines at the end of the laser ablation procedure, and applying; (i) the linear regression; and (ii) the Bland-Altman method for the data collected during the overall treatment.

## 2. Theoretical Background

### 2.1. Fiber Bragg Grating Sensors

An FBG consists of a periodic modulation of the refractive index of an optical fiber core along the direction of propagation. The exposition to UV light accomplishes this variation which will depend on the energy employed for the fabrication and on how it has been delivered (exposure pattern).

The described fabrication processes led to a grating which works as a wavelength selective mirror, reflecting a narrowband spectral component centered at the Bragg wavelength (*λ_B_*) where the maximum reflection occurs when satisfying the Bragg condition [37]:

(1) $$\lambda_{B}=2n_{eff}\Lambda .$$

The coefficient *n_eff_* is the effective refractive index of the fiber core over the exposed region and *Λ* is the period of the refractive index modulation (Grating period). According to Equation (1), the change of these two parameters affects *λ_B_*. These alterations can be caused by: (i) Mechanical tension of the fiber, leading to a displacement of *Λ* and a deviation in *n_eff_* due to the stress-optic effect; (ii) temperature variation affecting *n_eff_* through the thermo-optic effect and causing thermal expansion of the fiber (*Λ* shift). Hence, an FBG is sensitive to both strain variation (Δ*ε*) and temperature deviation (Δ*T*). A linear shift of the *λ_B_*, Δ*λ,* takes place when temperature variations are low or moderate [38],

(2) $$\Delta \lambda =2\left(\Lambda \frac{\partial n_{eff}}{\partial \varepsilon }+n_{eff}\frac{\partial \Lambda }{\partial \varepsilon }\right)\Delta \varepsilon +2\left(\Lambda \frac{\partial n_{eff}}{\partial T}+n_{eff}\frac{\partial \Lambda }{\partial T}\right)\Delta T$$

which can be reformulated as,

(3) $$\Delta \lambda =k_{\varepsilon }\varepsilon +k_{T}\Delta T$$

highlighting the strain sensitivity *k_ε_* (~1 pm/με) and temperature sensitivity $k_{T}$ (~10 pm/°C). Finally, the sensing mechanism of an FBG-based sensor relies on the detection of the Δ*λ* caused by variations of physical parameters (temperature, pressure, etc...). Assuming that no mechanical stress is applied to gratings, Equation (3) becomes,

(4) $$\Delta \lambda =k_{T}\Delta T$$

where the relationship between temperature and *λ_B_* is governed by $k_{T}$, which enclosed the thermal expansion coefficient of the fiber material and the thermo-optic coefficient.

FBGs sensitivity is constant in the range of interest of thermal ablation (i.e., from body temperature to 100 °C and above) and temperature measurements with 0.1 °C accuracy can be achieved. According to the most recent femtosecond point-by-point inscription technology, FBGs with a length down to 0.1 mm can be inscribed in polymer-coated optical fibers [39], which enables the fabrication of sensor arrays with enhanced sensing capacity. FBG sensors calibration is necessary to evaluate the temperature sensitivity. It can be accomplished through a controlled water bath or a thermostatic chamber; during controlled heating, the *λ_B_* of each FBG is recorded by a reference temperature sensor. The calibration curve and so $k_{T}$ is obtained by a linear fit of the data provided by all the FBGs.

### 2.2. Magnetic Resonance Thermometry

Temperature maps are computed from the temporal difference in phase images acquired with the GRE-EPI sequence. The direct proportionality relationship between the phase variation, Δφ, and the temperature change Δ*T* is,

(5) $$\Delta T=\;K\cdot \Delta \varphi =\;\frac{\varphi -\varphi_{REF}}{\gamma \cdot \alpha_{PRF}\cdot B_{0}\cdot TE}$$

**Figure 1:**
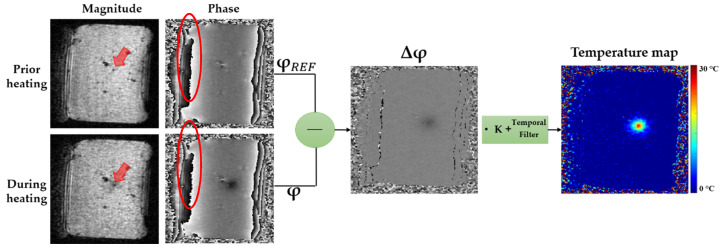
Computational process to reconstruct temperature maps using PRF thermometry method during agar gel laser ablation. Laser applicator is perpendicularly positioned respect to the imaging plane and can be depicted in the magnitude image (red arrow).

Δ φ = (φ − φ*_REF_*) is the phase shift, where φ is the current acquired phase image, φ*_REF_* is the phase of reference (baseline) image acquired before heating. The temperature map is then calculated from the subtracted phase Δφ multiplied by the constant factor *K* whose value depends on: *γ* (gyromagnetic ratio of ^1^H, equal to 42.58 MHz/T), *α_PRF_* (PRFS temperature coefficient, equal to −0.0094 ppm/°C for most aqueous tissues [40]), *B*_0_ (magnetic field strength) and TE (echo time of the MR sequence). Thus, the obtained temperature map displays the relative temperature changes compared to the baseline condition (corresponding to φ*_REF_* image). Figure 1 illustrates the basic principle of PRF thermometry method for a laser heating of a static agar gel. By subtracting a baseline reference phase image (acquired before the onset of temperature change) from phase images acquired, while the temperature changes, non-temperature related phase variations are removed. Numerous factors can lead to phase variations such as: Magnetic inhomogeneities of the *B*_0_ field or phase offsets between the various radiofrequency-receive or transmit Magnetic Resonance Imaging (MRI) coils. However, due to the restricted domain (−π to π) of the phase, local phase wraps corresponding to phase jumps of 2π occurred when the phase value overcomes −π or π. These phase wraps are evidenced in Figure 1 by the red ellipsoids. When the wraps are spatially shifting (due to phase uncertainty or *B*_0_ shift) they induce temperature artifacts due to the imperfect subtraction of the baseline phase image. Temporal filters can be used to remove these rapidly varying phase wraps artifacts from the final temperature map [41].

Under specific assumptions [23], the noise level of the phase images can be derived from the *SNR* of the magnitude-reconstructed image and the uncertainty of the temperature measurement can be expressed as follows,

(6) $$\sigma_{\Delta T}=\frac{\sqrt{2}\;\cdot K}{SNR}$$

where *SNR* is the signal-to-noise ratio of the magnitude image, here defined as *SNR* = A/*σ*. With A the signal intensity and *σ* the noise. The compromise between a low *K* value and a high SNR allows determining the theoretical optimal *TE* value for the thermometry of *TE* = T_2_* [40].

## 3. Materials and Methods

### 3.1. Experimental Setup

#### 3.1.1. Agar Gel

The investigation of MRTI performances was performed on a 3% agar gel phantom. Agar gel is widely adopted in MRI studies due to its similarity with biological soft tissue properties. In particular, for the PRF technique, its suitability has been demonstrated in a wide temperature range and the correlation between PRF shift and temperature change in agar gel is approximately linear [42]. The gel was prepared adding 30 g of noble agarose in 1 L of milli-Q water and then heating and blending the solution with a magnetic stirrer hot plate until the agarose was completely dissolved. Overboiling of the solution was avoided as the evaporation may alter the final percentage of agarose in the gel. Afterwards, the solution was placed at 4 °C for the solidification and for preventing the gel from drying out.

#### 3.1.2. Customized Box

**Figure 2:**
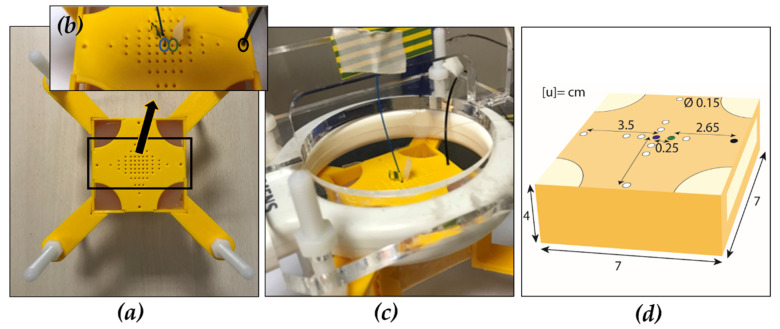
(**a**) Top view of 3D printed box (7 × 7 × 4 cm^3^) with agar gel phantom inside. Box tap pattern aimed at controlling the relative distance among the laser optical applicator and the FBGs array is showed. Laser optical applicator (blue circle), FBGs array (green circle), and fluoroptic sensor (black circle) arrangement in the box (**b**), (**c**) and their relative distances (**d**). The FBGs hole center (green spot in (**d**)) is at 0.25 cm from the central hole center (blue spot in (**d**)) and 2.65 cm from the center of the Luxtron sensor hole (black spot in (**d**)).

An ad hoc three-dimensional (3D) printed box in polylactic acid was fabricated to control the relative positioning of the FBGs and the laser fiber inside the phantom. The gel phantom perfectly fitted inside the parallelepiped shaped box (Figure 2a). The setup accurately arranged the laser optical applicator and the FBGs inside the gel, and adjustment of the relative distance among them due to the pattern shown in Figure 2a, characterizing both the top and bottom surfaces. The central hole is meant for housing the laser applicator, all the others offer a wide range of possible collocation for the FBGs array. The farthest holes from the center were aimed at the insertion in the gel of an MRI-compatible optical probe (Luxtron probe, LumaSense Technologies, Santa Clara, CA, USA) for measuring the initial temperature (about 22 °C). Detail in Figure 2b,c show how the fluoroptic sensor, FBGs array, and laser applicator were arranged within the pattern on the box top side. While, Figure 2d shows their relative distances. The fiber was inserted orthogonally to the phantom and parallel to the laser applicator which was about 10 mm deep in the gel. The 3D-printed box was then inserted inside a bigger holder, filled with water, in order to perform the experiments with the agar gel immersed in water leading to two major benefits. The surrounding water is responsible for keeping the agar gel hydrated and it prevents image distortion at the edges of the object, smoothing the drastic change in magnetic susceptibility thanks to agarose–water interface instead of agarose–air one.

#### 3.1.3. Laser

Laser ablation was performed by laser diode (975 nm, LuOcean Mini 4, Lumics, Berlin, Germany) placed immediately outside from the MR room, delivering the radiation to an MR-compatible fiber applicator (core of diameter 365 μm, THORLABS, Dachau, Germany). It operated with a power of 1.1 W for 3.5 min on the same 3% agar gel.

#### 3.1.4. Fiber Bragg Grating Sensors Setup

A millimeter spatial resolution was achieved during experiments by employing a 24.9-mm fiber sensor containing 25 uniformly distributed FBGs. Each FBG has an individual resonant wavelength laying in 1460–1620 nm spectral region, the length of 0.9 mm, and the center-to-center distance to the neighboring FBG of 1 mm. A total sensing length of ~25 mm was set to be reasonable to retrieve information of the area subjected to the temperature increase. Indeed, in the laser ablation scenario, a typical diameter of 15 mm is reached in the treatment [34]. The FBG array is housed in SM1500(9/125)P fiber (Fibercore Ltd., Southampton, UK), which is coated by polyimide and has a reduced diameter (~155 μm), thus providing superior durability of the sensor as compared to standard solutions based on acrylate-coated fibers. The sensor was fabricated with the femtosecond point-by-point inscription technology using the in-house facilities [39]. The inscription setup is based on Pharos 6W (Light Conversion, Vilnius, Lithuania) femtosecond laser system producing pulses with a wavelength of 1026 nm and duration of 232 fs and ABL1000 (Aerotech Inc, Pittsburgh, PA, USA) air-bearing linear stage enabling high-precision traveling of a fiber, while the inscription process. Tight focusing of femtosecond pulses into the core region of a fiber is carried out using a high-aperture objective. Modulation of the refractive index along the fiber is achieved by moving it with a predefined velocity profile and simultaneous irradiation with laser pulse sequence (*f* = 1 kHz). In our case, the velocity of a fiber was *v_i_* ~ 1 mm/s, and Bragg resonance wavelengths are determined as *λ_B,i_* = *2n_eff_Λ_i_* = *2n_eff_v_i_/*(*mf*), where *m* = 2 is an order of FBG.

The FBG array was placed at the same quote of the laser fiber tip (10 mm deep into the gel). An optical spectrum analyzer, HYPERION (Optical Sensing Instrument si155, Micron Optics Atlanta, GA, USA), with a sampling frequency of 100 Hz, was used to interrogate FBGs and to monitor *λ_B_* during experiments. A laptop was employed to collect data from the optical spectrum analyzer.

#### 3.1.5. MR Scanner and Sequences

The heating process was monitored by 1.5 T MR scanner (MAGNETOM Avanto, Siemens Healthcare, Erlangen, Germany) equipped with one standard loop coil positioned above the phantom and an 18 channels body coil positioned under the sample. A single-shot GRE-EPI pulse sequence was used with an acceleration factor of 2 and GRAPPA reconstruction, as well as with a 75% partial Fourier acquisition allowing to reach an echo time of 23 ms and a bandwidth of 666 Hz in the phase encoding direction. During all the tests, three slices were acquired in sagittal orientation. Saturation slabs were positioned along the field of view in the phase encoding direction to avoid aliasing of the water signal surrounding the gel. GRE-EPI sequence parameters are reported in Table 1, the matrix size values are the ones before the zero-filling process. In-plane zero filling (factor 2) was applied prior Fourier transform of MR data, resulting in a reconstructed voxel size of 0.5 × 0.5 × 2 mm^3^.

The parameters were experimentally tuned to provide sufficient image quality while ensuring rapid monitoring of temperature changes in the gel. To this end, both T_1_ and T_2_* of the agar gel were evaluated (T_1_ ≈ 1.8 s and T_2_* ≈ 50 ms), allowing to calculate the optimal flip angle corresponding to the Ernst angle $\alpha_{E\;}$ [43], calculated according to Equation (7):

(7) $$\alpha_{E\;}=\arccos\left(e^{-\frac{TR}{T1}}\right).$$

However, this flip angle value is optimal at room temperature only; to take into account the T_1_ increase due to the heating [44] and avoid signal saturation the flip angle was reduced by 30%. The minimum *TE* value of 23 ms was used instead of the theoretical optimal one (*TE* = T_2_*) of 50 ms to get closer to in vivo condition, as well as minimizing the susceptibility artifacts rising from the gel-air interfaces [19].

**Figure 3:**
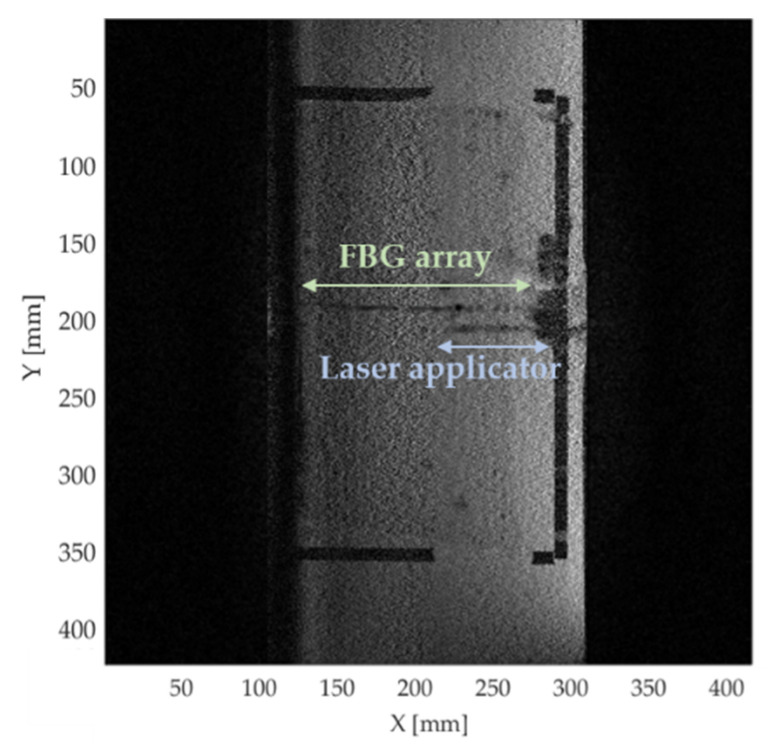
High resolution magnitude image obtained by the 2D GRE sequence. The fiber housing FBG is detectable thanks to a 0.23 × 0.23 mm^2^ in-plane resolution and the high SNR. Both the sensing fiber and the laser are visible on the same image.

A high in-plane resolution gradient echo (GRE) sequence was used to determine accurately the sensors’ localization. A high-resolution two-dimensional (2D) GRE sequence whose acquisition parameters are reported in Table 2 was used for this purpose. The resulting image is showed in Figure 3, in which both the laser applicator and the fiber housing FBGs are visible.

Identical field of views and position of the stack of slices were used for high-resolution GRE and GRE-EPI sequences to easily retrieve the fiber position on both images:

(8) $${\left(\mathrm{Pixel}\_\mathrm{Number}\right)}_{\mathrm{EPI}}\;=\frac{\left(\mathrm{Pixel}\_\mathrm{Number}\right){\;}_{\mathrm{GRE}}\cdot {\left(\mathrm{Matrixe}\_\mathrm{Size}\right)}_{\mathrm{EPI}}}{{\left(\mathrm{Matrixe}\_\mathrm{Size}\right)}_{\mathrm{GRE}}}.$$

**Figure 4:**
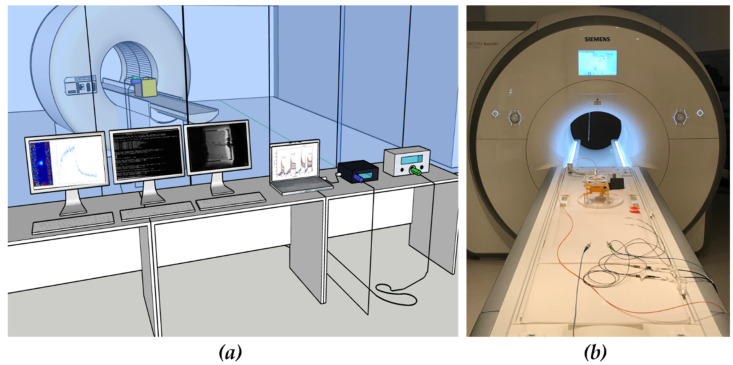
(**a**) Experimental setup for performing laser ablation on an agar gel phantom while monitoring the temperature with FBG sensors and MRTI. The laser system and spectrum analyzer are placed in the control room from which is possible to see the MR scanner. The operator console and real time reconstruction pipeline are also represented, the temperature map is finally displayed on dedicated thermometry software; (**b**) photograph of setup arrangement within the Magnetic Resonance scanner room.

Starting from the acquired raw data, images were online reconstructed by Gadgetron framework [45]. Then, temperature images were displayed on dedicated thermometry software (Thermoguide, IGT, Pessac, France) thanks to a remote host computer. All the data were streamed through TCP/IP on a sub-second timescale. The pipeline runs on a Core i7-4790 processor (3.6 GHz, four cores, INTEL Santa Clara, CA, USA) operating under Linux. Figure 4a offers a general view of the entire experimental setup. In the control room, the spectrum analyzer and the laser system are visible together with the operator console, and the computers are dedicated to running the Gadgetron framework and to real time thermal maps monitoring. A view of how the setup is arranged inside the scanner is given in Figure 4b, the box is then inserted within the water-filled container.

### 3.2. MRTI Analysis

The MRTI analysis was performed off-line on Matlab R2018a^®^. The reference phase image φ_REF_ was obtained by averaging 28 single-shot GRE-EPI images acquired before heating. The temporal filtering of the temperature maps was performed by symmetrical Butterworth filter with a cutting frequency of 0.05 Hz. No spatial filtering was used. However, the temperature maps have been masked out to remove the temperature values in pixels with low MRI magnitude signal. This mask is obtained by a thresholding operation based on the magnitude images with a threshold of 0.2 times the maximum amplitude (empirically determined). To keep the points corresponding to small signal drops within the gel and remove the overall edges, morphological closing and morphological erosion on the mask were performed with disk shape patch with radius 5, and 3 pixels, respectively. In the temperature maps displayed in this article, only the temperature value above +1 °C are shown for visibility.

To evaluate the MRTI precision using a standard approach, temperature uncertainty was firstly estimated using Equation (6). The noise 𝜎 was considered equal to the standard deviation in a region outside the agar gel. The SNR and the consequent $\sigma_{\Delta T}$ were then calculated in three different 3 pixels-regions of interest (ROIs) respectively placed in; (1) the area closest to the laser (highly heated) where a ΔT~25 °C is measured; (2) an area experiencing a temperature gradient of ~10 °C (moderately heated); and (3) an area outside the target ablated one (non-heated). In each ROI, $\;\sigma_{\Delta T}$ was extracted before and at the end of the ablated procedure, and during the cooling phase.

### 3.3. Data Analysis for FBGs and MRTI Comparison

As a first step, the comparison between FBGs and MRTI measurements was performed extracting the temperature profiles along a line at the end of the laser emission. For each FBG punctual measurement, the last ten seconds before the end of the heating were averaged (see Figure 7). The result of the averaging operation consists of twenty-five values representative of the temperature plateau, one for each grating. These points were interpolated in MATLAB^®^ to obtain a temperature profile matching FBGs sensors position. A one-dimension ROI composed of 50 pixels (fiber sensing length is 24.9 mm) was selected on the temperature map from the EPI image. The last 10 images, which again correspond to the 10 s at the end of the ablation (Figure 7), were averaged, in order to obtain a single image representative of the temperature plateau. The ROI was placed in correspondence of the FBG array on the image, and the optimal MRTI temperature space line was finally compared with the measurements from FBGs. The two temperature spatial profiles were quantitative compared with the RMSE parameter.

Furthermore, the linear regression and the Bland-Altman method were performed to compare the MR-based approach to the reference one based on FBGs using the data collected during the laser ablation of the agarose phantom. The samples studied for the comparison were taken from the 25 FBGs readings as regard as the reference data. On the other hand, for the MRTI measurements, the one-dimensional (1D) ROI, composed of 50 pixels used for obtaining the space temperature lines, was again considered. However, for the comparison with the 25 FBGs measurements, the 50 pixels were averaged in pairs of two because each pixel is 0.5 mm side while the FBG sensing length is 0.9 mm. For the linear regression approach, the heating and cooling phases were separately analyzed. Concerning the Bland-Altman, data for all the 5 tests acquired during the heating phase were combined to obtain a single mean of difference (MOD) value, employed as an index of the accuracy of the investigated method, and overall limits of agreements (LOA) representing the limits, including 95% of differences between the two measurement methods [46].

## 4. Results

### 4.1. Analysis of MRTI during Laser Heating

**Figure 5:**
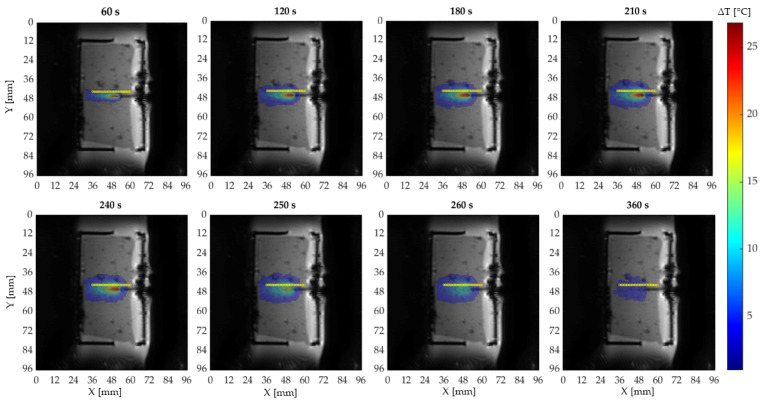
Temperature distribution in eight different time instants: The first five images are acquired during the ablation and the last three during the cooling period. The yellow markers specify the FBGs position.

Temperature maps obtained during the laser ablation for the agar gel are shown in Figure 5. Here, yellow dots show 25 FBGs positions respect to the ablated area.

### 4.2. MRTI Precision

**Figure 6:**
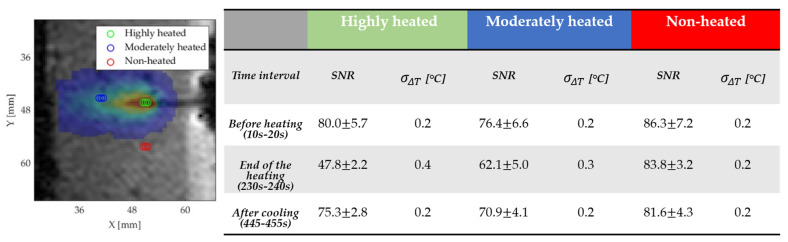
SNR and uncertainty $\sigma_{\Delta T}$ results for the three ROIs shown in the figure on the in the three-time intervals (see Figure 7).

Figure 6 shows the SNR and correspondent uncertainty $\sigma_{\Delta T}$ values obtained in three-time intervals. Data are reported for each ROI of 3 pixels, whose positions are also visible in Figure 6, and represent the values achieved as average among the 5 tests.

Uncertainty values are comprised between 0.2 °C, in the areas not subjected to the laser treatment, and 0.4 °C, close to the laser tip. These results show the dependence of the SNR to the temperature increase due to the T_1_ increase and the intra-voxel dephasing due to the intra voxel temperature gradients. The temperature increase leads to a signal drop followed by an SNR decrease for equal noise.

### 4.3. Comparison between FBGs and MRTI Temperature Values

**Figure 7:**
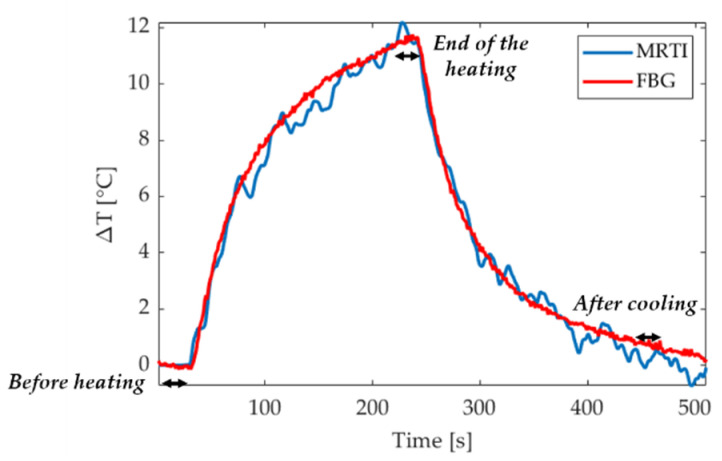
An example of temperature evolution in time for the FBG (blue) and the MRTI (red). These data refer to one out of five different experiments. Time intervals used for the definition of the MRTI precision are also highlighted in the graph with black arrows.

**Figure 8:**
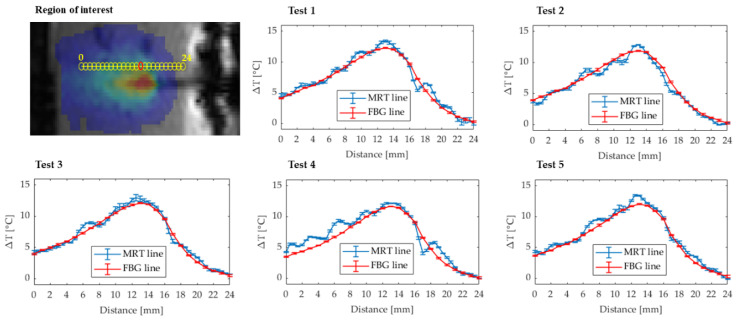
ROI in the MRTI images for the analysis and comparison between FBG and MRTI space temperature lines for 5 tests.

Temperature profiles measured by one FBG and in the corresponding pixel is reported for one test in Figure 7 (the position of this pixel is indicated in red color in Figure 8). On the other hand, Figure 8 reports a zoom for the MRTI image acquired in the temperature plateau showing the ROI of 50 pixels and the comparison between the temperature profiles measured by the FBGs and the MRTI from the five tests performed. The error bars represent the standard deviation associated with the averaging process over the last 10 s before the end of the ablation (temperature plateau). The standard deviation values obtained for MRTI measurements range between 0.05 °C and 0.5 °C, whereas the standard deviation obtained with FBGs never exceeds 0.1 °C. The volume heated by the laser source has an ellipsoidal shape and the heating propagates predominantly along the laser axis as clearly evidenced in Figure 5. Therefore, the temperature profile measured by the FBG in the specific configuration is not symmetrical with respect to the laser applicator tip: The FBGs downstream of the laser tip undergo higher temperature increase then the FBGs at the same distance but in the other direction. However, the 25 measurements show evident convergence along the whole distance considered for all the tests. RMSE values for the 5 tests are: 0.7, 0.7, 0.6, 1.2, and 0.7 °C, respectively.

**Figure 9:**
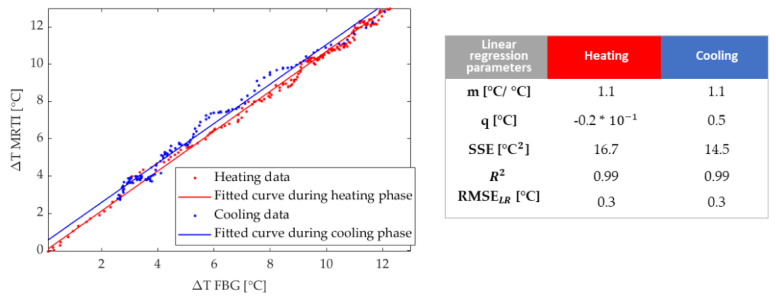
Linear regressions and their parameters for heating (red) and cooling (blue) phases for the Test 1. Fitted curves are plotted together with the data collected during the overall procedure.

A more quantitative comparison between FBG and MRTI temperature values was also performed by applying a linear regression for the data collected during the heating and cooling separately. Fitting curves and parameters are provided in Figure 9 and clearly show comparable behaviors in the heating and cooling phases. The root mean square error for the linear regression (RMSE_LR_) in this case represents the fitting error again comparing FBG and MRTI collected temperature values and providing an estimation of the MRTI accuracy based on the FBG sensors.

**Figure 10:**
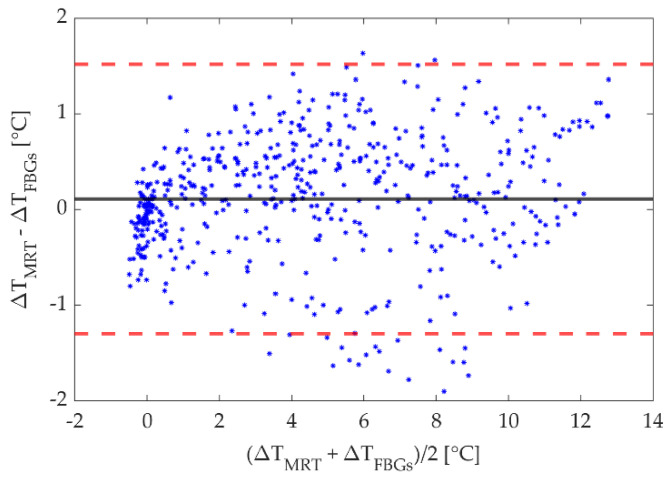
Bland-Altman analysis results for measurements performed on agar gel phantom. The LOA (dotted red lines) and the MOD (black line) are shown.

Finally, the Bland-Altman plot is shown in Figure 10. The dotted red lines and the black continuous line represent the LOA and the MOD respectively. Values are 0.1 °C and 1.5/−1.3 °C for MOD, and LOA, respectively.

## 5. Discussion

This work assessed the performance of an ultra-fast single shot GRE-EPI sequence, during laser ablation, on a 3% agar gel using several approaches. Uncertainty estimation through the SNR calculation depends on temperature temporal evolution. Indeed, $\sigma_{\Delta T}$ was found to be around 0.4 °C where the highest temperature gradient is measured and ~0.2 °C in the regions not subjected to temperature increase. In [14], similar values ranging from 0.2 to 0.4 °C are found. In this case, the temperature precision is measured as temporal standard deviation in a non-heated ROI and for eight different protocols including 2D GRE and 3D EPI during phantom laser therapy. Also in [22], the SNR was measured in a non-heated region and the lower value of 67 ± 3 for the EPI sequence was obtained for a phantom undergoing laser ablation and subjected to a temperature higher than the ones used in this work (around 75 °C). Conversely, our work takes into account the MR signal changing with temperature, investigating MRTI precision during the procedure in the three different areas. Nevertheless, SNR drop caused by temperature increase leads to uncertainty values always lower than 0.4 °C. Further limits of the use of this approach in our case study are found in the not optimal *TE* (≠T2*) set and in the SNR computation without considering parallel imaging use.

The drawbacks we highlight for the SNR-based precision estimation encourage the use of reliable temperature sensors for assessing the MRTI performances. Previous groups have already performed validation for GRE-EPI fast MRTI using reference sensors. Cernicanu et al. tested GRE-EPI with two different echo train lengths (ETL) during an radiofrequency ablation using four optical temperature probes as reference sensors (accuracy of ±0.5 °C and single point measurement) [18]. The Bland-Altman analysis implemented for the phantom validation showed values <0.4 °C and <1.4 °C, for MOD, and LOA respectively, during the heating phase for the two GRE-EPI sequences. Other research groups investigated the use of the GRE-EPI sequence for monitoring laser ablation procedure. For instance, Bazrafshan et al. tested both PRF and T_1_ sequences in the laser ablation scenario for a liver-equivalent phantom [22]. They found out that the GRE-EPI sequence is more appropriate for temperature monitoring in the laser ablation panorama in terms of higher precision and accuracy (MOD of 0.2 °C and LOA of 3.7/−3.2 °C for the GRE-EPI sequence versus 0.3 °C and 3.5/−2.9 °C for FLASH sequence) using two fiber-optic temperature probes. In [14], the accuracy of the set of 2D GRE and 3D EPI protocols was calculated also inserting a single fiber optic sensor as reference temperature. Comparison results, this time, were expressed as RMSE_LR_ and mean difference (0.5 °C, and <0.3 °C, respectively) for three 3D EPI protocols. One challenge highlighted in the analysis was that the fiber probe could be affected by the laser light. For this reason, the analysis was performed for temperature values acquired in a point far enough form the laser tip. The use of FBGs allows overcoming this issue giving the chance of acquiring temperature also at 1 mm distance from the laser source and in multiple spatial points of the target undergoing the procedure. In our study, the estimated MOD (around 0.1 °C) and the RMSE_LR_ (0.3 for both the heating and the cooling phases) agree with previous studies and satisfy the required precision for proper temperature monitoring during thermal therapies (1–2 °C). On the other hand, considering the results for LOA from literature, and the one obtained in this analysis (~1.5/−1.3 °C), they show different values demonstrating a strong dependence, among others, with specific sequence settings, image processing performed and spatial resolution obtained. In the present work, we deliberately chose to obtain high-resolution temperature images (reconstructed voxel size of 0.5 × 0.5 × 2 mm^3^) to minimize partial volume effect for better comparison with FBG measurements. Increasing the voxel size is expected to result in reduction of temperature standard deviation and thus lower LOA.

Our approach, based on ultra-dense 25 FBGs array with 1 FBG/mm sensing capacity, has been demonstrated to be particularly suitable for assessing MRTI performances, also in comparison with previous studies in which the number of fiber optic sensors, the measurement points, and the spatial resolution were limited by the technology [18,47]. The proposed sensing system allowed the comparison on the sensor-pixel scale, providing a maximum RMSE parameter of ~1.2 °C at the end of ablation when the maximum temperature is reached and temperature control is necessary. Whether for a homogeneous mean the possibility of having several measurement points has given the chance of retrieving more reliable GRE-EPI sequence performances, an increasing number of punctual temperature information is, instead, necessary for non-homogeneous tissues. For living tissues undergoing thermal procedures, indeed, the local differences in heat conduction necessitate a spatial-dependent MRT performance assessment. This is even more true in the laser ablation and high-intensity focused ultrasound therapies, where high temperature gradients are reached in a few millimeters distance [31]. The potential of measuring temperature in several points of the tissue, thus, reconstructing a 3D spatial temperature map with 0.1 °C accuracy holds huge opportunities in the MRTI scenario. These 1 mm-spatially resolved temperature measurements can support the correction of artifacts affecting image quality and proved to be convenient for MRTI calibration. Therefore, future work could be addressed to investigate MRTI performances for ex vivo and in vivo tissues towards the implementation in the clinical scenario. In addition, due to a high resolution 2D GRE sequence in this analysis was possible to visualize both laser and sensing fiber allowing a direct pixel-FBG comparison for temperature values. Final estimated results benefit from such procedure, not requiring any averaging process among pixels in specific regions of interest. This mentioned process, indeed, can suffer for information losses or wrong data manipulation.

## 6. Conclusions

In conclusion, in this work, the performance evaluation for GRE-EPI acquisitions, with both SNR-based approach and FBGs reference temperatures, was performed. The agreement between MRTI and the optical temperature measurement is valid for the rapid GRE-EPI so proving that the use of such a fast sequence does not introduce systematic error affecting temperature accuracy. EPI sequence parameters, chosen to fulfill the requirements for temperature monitoring (in-plane resolution of 1 × 1 mm^2^ and a time resolution of 1 s) and found performances ($\sigma_{\Delta T}\leq$ 0.4 °C, RMSE_LR_ ≤ 0.3 °C and MOD around 0.1 °C) make this sequence adapted for temperature monitoring of laser ablation. Further studies are required to extend this analysis for in vivo tissues. Nevertheless, potentialities hold by GRE-EPI, as well as FBGs capabilities in supporting MRTI, make already plausible the chance of using this sequence in the clinical scenario for laser ablation real-time temperature guidance.

## Figures and Tables

**Table 1:** GRE-EPI sequence parameters used for MRTI during laser ablation on agar gel.

Parameters	Experiments
Field of view [mm]	96 × 96
Matrix size [pixel]	96 × 96
Spatial resolution [mm^3^]	1 × 1 × 2
Pixel size after zero filling [mm^3^]	0.5 × 0.5 × 2
TR [ms]	1000
TE [ms]	23
Flip angle [°]	45
Bandwidth in read direction [Hz/pixel]	1086
Bandwidth in phase direction [Hz]	666
Total experiment duration [s]	510

**Table 2:** 2D GRE sequence parameters used for High Resolution imaging.

Parameters	High Resolution 2D GRE
Field of view [mm]	96 × 96
Matrix size [pixel]	416 × 416
Spatial resolution [mm^3^]	0.23 × 0.23 × 2
TR [ms]	100
TE [ms]	9.7
Flip angle [°]	35
Bandwidth [Hz/pixel]	100
TA [s]	703
Number of slices	3
GRAPPA acceleration	2
Number of averages	32
